# Absolute quantification of endogenous angiotensin II levels in human plasma using ESI-LC-MS/MS

**DOI:** 10.1186/1559-0275-11-37

**Published:** 2014-10-27

**Authors:** Anna Schulz, Joachim Jankowski, Walter Zidek, Vera Jankowski

**Affiliations:** Institute for Molecular Cardiovascular Research (IMCAR), RWTH Aachen University, University Hospital, Pauwelsstrasse 30, D-52074 Aachen, Germany; Charité-Universitätsmedizin Berlin (CBF), Medizinische Klinik IV, Berlin, Germany

## Abstract

**Background:**

Angiotensin II acts as a peptide hormone and component of renin-angiotensin- system (RAS) regulating the blood pressure, and seems to be involved in renal and vascular disorders. There is no reliable quantification method for angiotensin II available until now and the angiotensin II plasma levels described in the literature are correspondingly strongly divergent. Therefore, we developed and validated a sensitive, selective and reliable LC-ESI-MS/MS method for absolute quantification of angiotensin II concentration in human plasma based on the AQUA strategy.

**Methods:**

Plasma samples were extracted using MAX Oasis cartridges and were subjected to a further immunoaffinity-purification using immobilized anti-angiotensin II antibodies in order to isolate endogenous angiotensin II. Stable isotope (^13^C- and ^15^ N-) labeled angiotensin II was used as an internal standard. The fractionated samples were analysed using LC-ESI-MS/MS.

**Results:**

The calibration curve was established in plasma in the concentration range 6–240 pM (r^2^ > 0.999). The developed and validated method was successfully applied for quantification of endogenous angiotensin II in human plasma of healthy volunteers and chronic kidney disease (CKD-5D) patients. The mean plasma angiotensin II levels were found to be 18.4 ± 3.3 pM in healthy subjects and 64.5 ± 32.4 pM in CKD-5D patients (each n =9).

**Conclusion:**

The LC-ESI-MS/MS-based method for quantification of angiotensin II levels in human plasma was successfully evaluated within the study. This method is applicable for clinical applications aiming at the validation of the impact of highly physiologically and pathophysiologically active angiotensin II.

## Introduction

The renin-angiotensin system (RAS) is essential for the maintenance of blood pressure and fluid balance [1]. The key component of the RAS is the peptide hormone angiotensin II, which was first isolated in 1939 by Braun-Menendez and Fasciolo from the renal blood of dogs [2]. This octapeptide with the amino acid sequence Asp-Arg-Val-Tyr-Ile-His-Pro-Phe is one of the most potent vasoconstrictors known until now. Angiotensin II has a vasoconstrictory effect on the efferent arterioles of the kidney. This leads to an increase of the intraglomerular vascular resistance, which in turn causes an increased filtration pressure. Besides the vasoregulatory effect of angiotensin II, it has been demonstrated that angiotensin II is characterized by proinflammatory, profibrotic and growth stimulating properties [3]. Experimental and clinical studies have shown angiotensin II affects the progression of chronic kidney disease (CKD) [4]. Angiotensin II causes an increased intraglomerular pressure and hyperfiltration and thus accelerates renal failure. In addition, angiotensin II affects podocyte function and thus may lead to proteinuria. This in turn may cause tubulointerstitial inflammation. Finally, these angiotensin II-mediated changes result in the histological picture of tubulointerstitial fibrosis and glomerulosclerosis [5]. Currently, the routine quantification of angiotensin II is performed using antibody-based fluorescence assays. However, it has been shown that commercially available monoclonal anti-angiotensin II-antibodies are characterized by high cross reactivity because of sequence homologies of different angiotensin peptides [6, 7]. Most likely these methodological difficulties may lead to the extremely divergent angiotensin II levels determined in different studies. These vary in healthy subjects in the range of 3–85 pM [6, 8–12].

Mass spectrometry-based methods, using stable isotope-labeled peptides, allow absolute quantification (AQUA) in low femtomole range and are especially useful when no analyte-free matrix is available [13, 14]. Therefore, the goal of this study was the development of a highly selective, sensitive, accurate and precise method for absolute quantification of endogenous angiotensin II levels in human plasma, based on combination of the immunoaffinity-purification and mass spectrometric detection by using stable isotope (^13^C- and ^15^ N-) labeled angiotensin II as an internal standard.

## Materials and methods

### Reagents and chemicals

Standard angiotensin II was purchased from Sigma-Aldrich (Hamburg, Germany). Stable isotope labeled angiotensin II (^13^C and ^15^ N labeled arginine) was synthesized by Campro Scientific GmbH (Berlin, Germany). MS-grade water and acetonitrile were purchased from Thermo Fisher Scientific (Ulm, Germany). MS-grade formic acid, CN-Br activated sepharose resin beads, protease inhibitor cocktail and PBS were purchased from Sigma-Aldrich (Hamburg, Germany). Monoclonal anti-angiotensin II-antibodies were purchased from Bertin Pharma (Montigny le Bretonneux, France). Low protein-bind tubes were obtained from Eppendorf (Hamburg, Germany). The Oasis MAX extraction cartridges were purchased from Waters (Eschborn, Germany).

### Immobilisation of the anti-angiotensin II-antibody

Anti-angiotensin II antibody (80 μg) was diluted in 1 ml of coupling buffer (0.5 M NaCl, 0.1 M NaHCO_3_, pH 8.3) prior to immobilization. Three batches of cyanogen bromide activated sepharose resin (500 μl) were swollen in 10 ml of 1 mM cold HCl for 1 hour. The resin was then washed three times with 10 ml of 1 mM HCl and three times with 10 ml of coupling buffer. The diluted antibody was immediately transferred to the washed resin. The immobilization was carried out 1 h at room temperature on an overhead rotor. After the immobilization the unbound substrate was washed out three times using 1.5 ml of blocking buffer (1 M NaCl, 0.05 M glycine, pH 3.5). The supernatant was removed and the unbound reactive groups of the sepharose resin were blocked using blocking buffer 2 h at room temperature. After the blocking procedure the resin was washed alternately using 1.5 ml of washing buffer 1 (0.5 M NaCl, 0.1 M NaCH_3_COO, pH 4) and 1.5 ml of washing buffer 2 (0.5 M NaCl, 0.1 M Tris, pH 8). The immobilized anti-angiotensin II-antibodies were stored in PBS containing 0.02% NaN_3_ at 4°C until use. Prior to use, the immobilized anti-angiotensin II-antibodies were washed five times using 1 ml phosphate buffered saline (PBS).

### Plasma collection

Peripheral blood (5 ml) from nine healthy volunteers and nine CKD patients undergoing dialysis (CKD-5D) was drawn from the cubital vein and was collected in tubes containing K_2_-EDTA (1.8 mg/ml). 50 μl of protease inhibitor cocktail was added immediately after blood sampling. The blood samples were centrifuged at 2,500 *g* for 10 minutes to isolate plasma. The resulting plasma was prepared as 0.5 ml fractions and 100 fmol of the internal standard solution (stable isotope (^13^C- and ^15^ N-) labeled angiotensin II) were added to each sample. The samples were immediately frozen and stored at −20°C until further sample preparation.

### Preparation of calibration standards and quality control samples

The stock solution of the stable isotope (^13^C- and ^15^ N-) labeled angiotensin II as well as native angiotensin II were prepared at 100 μM in 1% formic acid in water/acetonitrile (50/50; v/v) and were stored at −20°C. Only low protein bind tubes and pipette tips were used, in order to minimize nonspecific adsorption of the diluted peptide. The preparation of the calibration samples was carried out by spiking stable isotope (^13^C- and ^15^ N-) labeled angiotensin II into plasma at concentration of 6, 15, 30, 60, 120, 240 pM in triplicate. The quality control samples (QC) were prepared in plasma with stable isotope (^13^C- and ^15^ N-) labeled angiotensin II at a concentration of 6 pM for the lower limit of quantification (LLOQ), at 30 pM for low quality control (LQC), at 120 pM for middle quality control (MQC) and at 240 pM for high quality control (HQC). The native angiotensin II was used as an internal standard and was added to the samples at a final concentration of 200 pM, which corresponds to the endogenous concentration of angiotensin II in previous studies as well in the present study revealed a wide range of the angiotensin II concentrations.

### Sample preparation

The calibration samples, quality control samples and plasma samples were prepared in the same approach. Oasis mixed-mode anion exchange and reversed phase (MAX) solid phase extraction (SPE) cartridges were used to remove high molecular plasma compounds and concentrate angiotensin II. 0.5 ml plasma were thawed and diluted with 0.5 ml of 4% phosphoric acid. The extraction cartridges were preconditioned with 1 ml acetonitrile followed by 1 ml LC-MS grade water. Next, the sample was loaded. The cartridge was washed with 1 ml 5% ammonium hydroxide followed by 1 ml 10% acetonitrile. The samples were eluted from the SPE cartridge with 1 ml 2% formic acid in 75% acetonitrile. The eluate was evaporated to dryness, reconstituted with 300 μl PBS and, if required, the pH was adjusted at 7.4 using 10 M sodium hydroxide. For further immunoaffinity purification of angiotensin II the extracted samples were incubated 1 hour at 4°C with 30 μl of immobilized anti-angiotensin II antibody. Next, the beads were washed two times using 500 μl PBS. In order to remove all salt the resin was washed two times using 500 μl LC-MS grade water. Angiotensin II was eluted three times using 40 μl of 0.1% formic acid directly into the glass vial and evaporated to dryness. The dried samples were reconstituted in 40 μl 0.1% formic acid and analyzed by ESI-LC-MS/MS.

### LC MS/MS analysis

All LC-MS/MS analysis were performed using an Agilent 1200 capillary-HPLC system (Germany) coupled to a HCT mass-spectrometer (Bruker, Bremen, Germany) with an electrospray ionisation (ESI) interface. A C18 Aq Zorbax column (150 × 0.5 mm; 5 μm) was used for separation. HyStar-software (Bruker, Bremen, Germany) was used for data acquisition and processing. The mobile phase consisted of 0.1% formic acid in water (mobile phase A) and 0.1% formic acid in acetonitrile (mobile phase B). The flow rate was set to 20 μl/min and the injection volume was 40 μl. The column temperature was maintained at 30°C. The plasma samples were separated using gradient given in the Table 1. The total time per run was 25 min. The mass-spectrometer was operated in the positive ion mode. The source temperature was set at 300°C. The nebuliser gas was set at 20 psi and the dry gas flow was 7 l/min. For the detection of angiotensin II, the MS/MS fragmentation mode was used. The accumulation time was 50 ms. The double charged parent ions of the native angiotensin II and the stable isotope (^13^C- and ^15^ N-) labeled angiotensin II were 523.8 m/z and 528.8 m/z, respectively. The fragmentation of these ions revealed for both peptides one dominant fragment ion and several low intense ions. The high intense dominant fragment ion was 784.4 m/z for the native angiotensin II and 794.4 m/z for the stable isotope (^13^C- and ^15^ N-) labeled angiotensin II. Therefore, the system was tuned and optimized for the transition 523.8 m/z - > 784.4 and 528.8 - > 794.4 for the detection of native angiotensin II and the internal standard, respectively. Therefore, the isolation window 526.3 ± 6 m/z was found to be optimal for the detection of both fragment ions. The isolation and the detection of the both ions were performed within the same scan in order to achieve a high precision and accuracy. For the MS/MS mode, helium was uses as the collision gas. For the quantification the extracted ions chromatograms for the ions 784.4 ± 0.5 m/z and 794.4 ± 0.5 m/z corresponding to the native and stable isotope (^13^C- and ^15^ N-) labeled angiotensin II were generated and intergraded. Data acquisition and interpretation was performed by using “Compass 1.3 Software” (Bruker, Bremen, Germany). Calculations including calibration curve regressions, sample concentrations values and statistics were performed by using “GraphPad Prism 5.0” software (GraphPad Software, San Diego, USA).

**Table 1:** **LC-gradient used for the separation of the plasma samples**

Time [min]	Eluent A [%]	Eluent B [%]
0	100	0
0.1	80	20
8	75	25
10	0	100
11	0	100
11.1	100	0
25	100	0

### Validation procedure

The newly developed method was extensively validated in terms of selectivity, linearity, intra- and inter-day precision and accuracy, matrix effects and recovery. All samples used during validation procedure were taken from plasma pool from healthy volunteers. All these samples were pooled before the sample preparation.

### Linearity of the calibration

The linearity of the method was determined by analysing of calibration standards (CS) prepared by spiking pooled plasma samples using stable isotope (^13^C- and ^15^ N-) labeled angiotensin II in triplicate. The CS contained 6, 15, 30, 60, 120 and 240 pM of stable isotope (^13^C- and ^15^ N-) labeled angiotensin II. The native angiotensin II was used as an internal standard. The extracted ions chromatograms for the ions 784.4 ± 0.5 m/z and 794.4 ± 0.5 m/z corresponding to the native and stable isotope (^13^C- and ^15^ N-) labeled angiotensin II, were generated and the analyte peaks intergraded. The concentration curve was constructed by plotting the peak-area ratio of stable isotope (^13^C- and ^15^ N-) labeled angiotensin II/native angiotensin II vs. concentration.

### Lower limit of quantification (LLOQ) and limit of detection (LOD)

The lower limit of quantification (LLOQ) is defined as the lowest point of the calibration curve with a precision of CV = 20%. The limit of detection is defined as an amount giving a signal three times higher than the noise.

### Precision and accuracy

The intra- and inter-day precisions and accuracies were investigated by using quality controls (QC) at 6, 15, 30, 60, 120 and 240 pM of stable isotope (^13^C- and ^15^ N-) labeled angiotensin II prepared in plasma. The analysis of the intra-day precision and accuracy was performed in triplicate on the same day; the inter-day precision and accuracy were performed on three separate days. The precision was calculated as the relative standard deviation of the replicates. Accuracy was calculated by comparison of the measured concentration of spiked analyte with expected concentrations.

### Matrix effects

**Figure 1:**
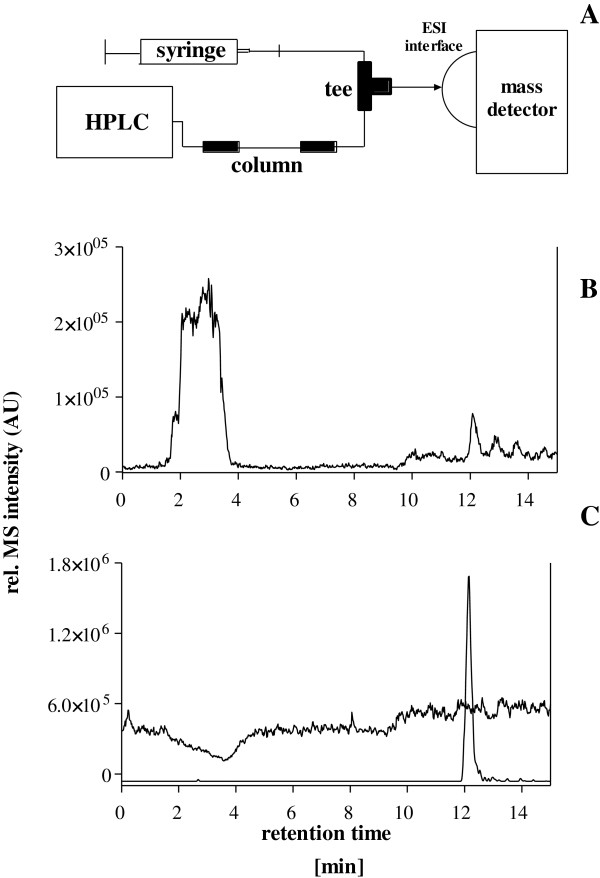
**Investigation of matrix effects by the post column infusion method defined by Bonfiglio et al. A**: Scheme of the post-column infusion system **B**: Total ion chromatogram **C**: Overlay chromatograms showing the matrix effects due to purified plasma on the detection of angiotensin II.

The matrix effects of the purified plasma were tested by using a method defined by Bonfiglio et al. The post-column infusion system was used for these experiments. The isolated sample was injected onto column and the gradient program was started, while angiotensin II was being infused post column (concentration 1 μM) at a flow rate 4 μl/min. The schematic post-column infusion system is shown in Figure 1A.

### Recovery

Recovery of angiotensin II was evaluated by comparison of peak area of the isolated stable isotope labeled angiotensin II with standard prepared in 0.1% formic acid at the concentrations of the quality controls representing 100% recovery. Recovery was analysed at four concentration levels corresponding to the quality controls in triplicates at each concentration level.

### Application of the method

The endogenous levels of angiotensin II in human plasma were tested in 9 healthy subjects and CKD-5D patients. Stable isotope (^13^C- and ^15^ N-) labeled angiotensin II at a concentration of 200 pM was used as an internal standard. The quantities of the native angiotensin II were calculated using the peak-area ratio of native angiotensin II/stable isotope (^13^C- and ^15^ N-) labeled angiotensin II (internal standard) multiplied by the absolute concentration of the internal standard (200 pM).

### Statistics

All results are presented as means ± SEM. Statistical significance was determined by use of Mann–Whitney-test.

## Results and discussion

**Figure 2:**
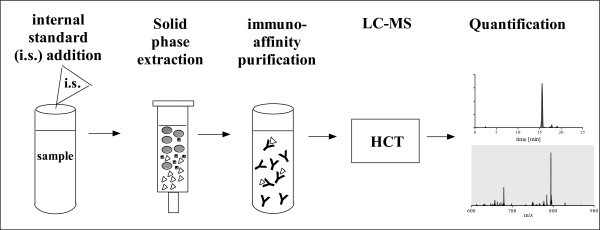
**Overview about sample preparation for the quantification of angiotensin II.**

**Figure 3:**
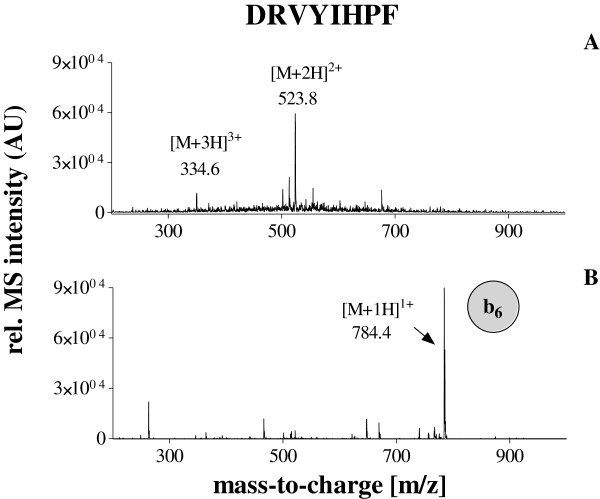
**Detection of native angiotensin II by ESI-MS A: positive full scan spectrum B: positive MS/MS spectrum of the parent ion 523.8 m/z.** The b_6_ fragment ion (784.4 m/z) was used for the quantification.

**Figure 4:**
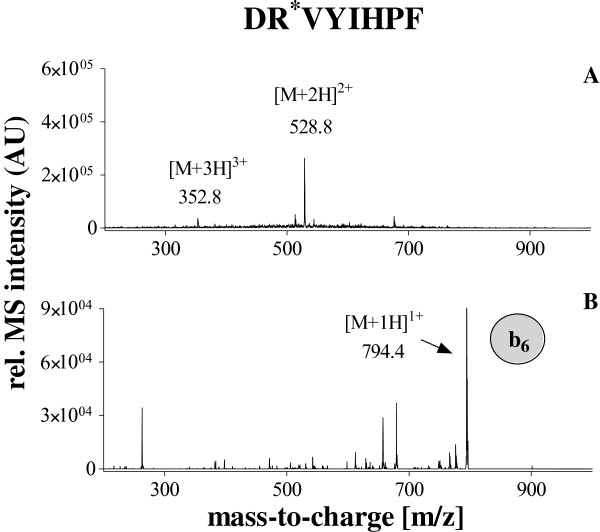
**Detection of stable isotope labeled angiotensin II by ESI-MS A: positive full scan spectrum B: positive MS/MS spectrum of the parent ion 528.8 m/z.** The b_6_ fragment ion (794.4 m/z) was used for the quantification.

In this study a new method was developed and validated for the absolute quantification of endogenous angiotensin II levels in human plasma, since recent studies demonstrated the insufficient specificity of the monoclonal antibodies using for the quantification of angiotensin II by commercial ELISA based assay [6, 7]. Plasma samples were extracted by using mixed-mode anion exchange and reversed phase (MAX) solid phase extraction (SPE) cartridges, followed by an immunoaffinity-purification. The purified samples were separated by using reversed-phase chromatography and analyzed by ESI-MS in fragmentation mode. The schematic overview of the sample preparation is shown in the Figure 2. Since the AQUA strategy was used for absolute quantification of the native angiotensin II in human plasma, we decided to isolate and fragment both the native and stable isotope labeled peptide in the same scan. Due to “scan to scan” variation of the electrospray ionisation, the detection of both peptides within the same scan improves the precision and accuracy of the method significantly. The fragmentation of both peptides revealed predominantly one intense ion corresponding to the b6-fragment of the angiotensin II, which could be stable detected independent of the concentration. Therefore, only this ion was used for the quantification. The isolation window of 526.3 ± 6 m/z was found to be optimal for the detection of both fragment ions: 784.4 m/z and 794.4 m/z of native angiotensin II and the internal standard respectively (Figures 3 and 4). Since the samples underwent extensive purification based on solid phase extraction and immunoaffinity-purification, the analysis of the resulting fractions revealed low background.

### Selectivity

The selectivity for the stable isotope (^13^C- and ^15^ N-) labeled angiotensin II was determined by comparison of quality controls and plasma samples. The retention time for angiotensin II was 12.1 min. No interfering peaks in the purified plasma samples were observed at the retention time of angiotensin II in the respective extracted ion chromatogram (data not shown).

### Detection limit and the calibration range of the stable isotope labeled (^13^C- and ^15^ N-) angiotensin II

**Figure 5:**
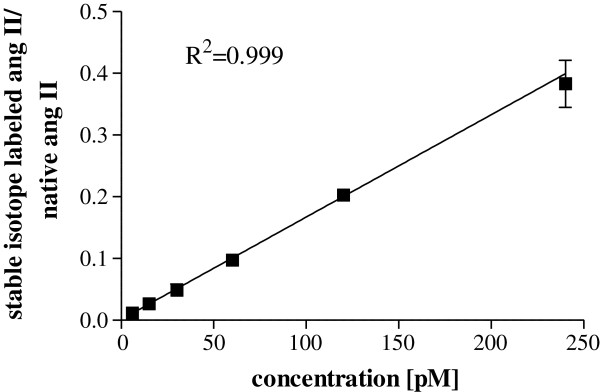
**Linear calibration curve for stable isotope labeled angiotensin II prepared in plasma (3–120 fmol on column; N = 3).**

The linearity range of the native angiotensin II was determined using plasma spiked with stable isotope (^13^C- and ^15^ N-) labeled angiotensin II. The six-point calibration curve of stable isotope (^13^C- and ^15^ N-) labeled angiotensin II showed a reliable reproducibility in the concentration range from 6 to 240 pM. The calibration curve was prepared by plotting peak-area ratio stable isotope (^13^C- and ^15^ N-) labeled angiotensin II/native angiotensin II vs. concentration and was fitted to linear regression with 1/SD^2^ weighting, which gave the best fit. The coefficient of determination r^2^ for validation was found to be 0.999 (Figure 5). The detection limit was determined to be 3 pM corresponding to the signal three times higher than the noise. The lower limit of quantification was determined to be 6 pM.

### Precision and accuracy

The accuracies and precisions for both intra- and inter-day measurements are presented in Table 2. The LLOD at at 3 pM in biologic matrix (plasma) is in accordance with the LOD-values in different biologic matrices (plasma, urine) recently described in the literature [15, 16]. The mean analytical intra- and inter-day precisions were found to be between 4.6% and 16.0%. The intra- and inter-day accuracies were between 91.5% and 112.5%.

**Table 2:** **Accuracy and precision of quality control samples**

			Intra-day (n = 3)		Inter-day (n = 9)	
	QC	Concentration (nM)	Accuracy (%)^1^	Precision (%)^2^	Accuracy (%)^1^	Precision (%)^2^
**Angiotensin II**	LLOQ	6	106.4	16.0	112.5	14.5
	LQC	30	96.6	6.6	91.5	8.1
	MQC	120	105.3	7.6	103.1	4.6
	HQC	240	92.7	7.8	94.6	9.0

^1^calculated as (mean determined amount/nominal amount × 100).

^2^calculated as % CV. (SD/mean) × 100.

### Matrix effects

The investigation of the matrix effects using post-column infusion of the native angiotensin II revealed no significant suppression, but slight enhancement of the angiotensin II signal around the specific retention time. Only a slight decrease in the mass peak intensity was detected at 4 min, which was most likely due to residual salts not retained by the column (Figure 1B). Figure 1C shows matrix effects chromatogram, overlaid by chromatogram of angiotensin II standards prepared in 0.1% formic acid to indicate the elution profile for the analyte over the infusion matrix effect baseline.

### Recovery rate

The mean recovery rate was found to be 34.0 ± 2.3% and the recovery rate was sufficient to quantify angiotensin II in the determined calibration range.

### Method application

**Figure 6:**
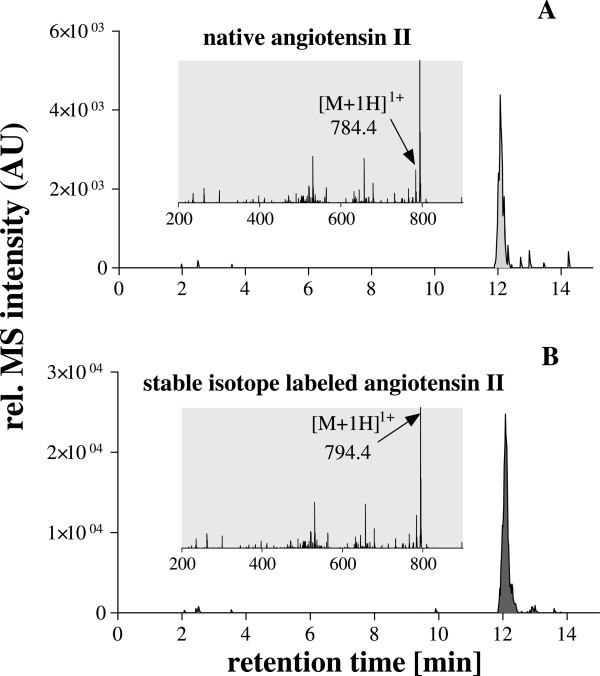
**Analysis of purified plasma sample from healthy volunteer by ESI-MS.** **A**: extracted ion chromatogram of the ion 784.4 ± 0.5 m/z corresponding to the native angiotensin II and a characteristic fragment spectrum with labeling of the b6-fragment (grey shaded area) **B**: extracted ion chromatogram of the ion 794.4 ± 0.5 m/z corresponding to the stable isotope labeled angiotensin II and a characteristic fragment spectrum with labeling of the b6-fragment (grey shaded area). Confirmation of the identity of the native ngiotensin II was performed by using both: retention time and fragment ion of co-eluting stable isotope ngiote ngiotensin II.

The new method was applied for quantification of angiotensin II levels in plasma from healthy volunteers and CKD-5D patients. The identity of the native angiotensin II was confirmed using both the retention time and the fragment ion from the co-eluting stable isotope labeled internal standard (Figure 6). The native angiotensin II was found in all plasma samples. Extracted ion chromatograms were generated and the analyte peaks integrated. The quantitative levels of the native angiotensin II were calculated using peak-area ratio of endogenous angiotensin II/stable isotope (^13^C- and ^15^ N-) labeled angiotensin II multiplied by the absolute concentration of the internal standard. The means plasma angiotensin II levels were found to be 18.4 ± 3.3 pM in healthy subjects and 64.5 ± 32.4 pM in CKD-5D patients (each n =9) and did not vary significantly between these both groups (Table 3). However, due to the small sample size the result might not be representative, thus of the impact of angiotensin II in the context of CKD should be verify within a large scale clinical study.

**Table 3:** **Endogenous ngiotensin II concentrations in 9 individual healthy subjects and CKD-5D patients**

	Endogenous angiotensin II concentrations [pM] in individual subjects									
Group	1	2	3	4	5	6	7	8	9	mean
Healthy subjects	30.2	9.7	19.2	9.5	10.2	33.5	24.9	22.7	6.6	18.5
CKD-5D patients	16.8	10.4	13.4	205.4	260.4	8.4	14.3	6.3	45.4	64.5

An extensively validated method for determination of the native angiotensin II in human plasma has been developed within this study. The AQUA strategy was used to successfully determine the absolute concentration of the native angiotensin II in plasma of healthy volunteers and CKD-5D patients. The new assay is highly selective, sensitive and reliable for the detection and quantification of angiotensin II between 6 and 240 pM. The analytical precisions and accuracies values are within the acceptable range.

### Ethical approval

This study was approved by the local ethic committee of the University Essen (approval number: 08-3817).

## References

[1] Zhuo JL, Ferrao FM, Zheng Y, Li XC (2013). New frontiers in the intrarenal renin-angiotensin system: a critical review of classical and new paradigms. Front Endocri (Lausanne).

[2] Braun-Menendez E, Fasciolo JC, Leloir LF, Muñoz JM (1940). The substance causing renal hypertension. J Physiol.

[3] Wolf G, Butzmann U, Wenzel UO (2003). The renin-angiotensin system and progression of renal disease: from hemodynamics to cell biology. Nephron Physiol.

[4] Siamopoulos KC, Kalaitzidis RG (2008). Inhibition of the renin-angiotensin system and chronic kidney disease. Int Urol Nephrol.

[5] Rodriguez-Iturbe B, Garcia Garcia G (2010). The role of tubulointerstitial inflammation in the progression of chronic renal failure. Nephron Clin Pract.

[6] Jankowski V, Vanholder R, van der Giet M, Tölle M, Karadogan S, Gobom J, Furkert J, Oksche A, Krause E, Tran TN, Tepel M, Schuchardt M, Schlüter H, Wiedon A, Beyermann M, Bader M, Todiras M, Zidek W, Jankowski J (2007). Mass-spectrometric identification of a novel angiotensin peptide in human plasma. Arterioscler Thromb Vasc Biol.

[7] Jankowski V, Tölle M, Santos RA, Günthner T, Krause E, Beyermann M, Welker P, Bader M, Pinheiro SV, Sampaio WO, Lautner R, Kretschmer A, van der Giet M, Zidek W, Jankowski J (2011). Angioprotectin: an angiotensin II-like peptide causing vasodilatory effects. FASEB J.

[8] Grzybowski J, Bilińska ZT, Janas J, Michalak E, Skwarek M, Ruzyłło W (2002). Level of angiotensin II and aldosterone in plasma is often elevated in patients with heart failure treated with converting enzyme inhibitors–preliminary results. Przegl Lek.

[9] Hermann K, von Eschenbach CE, von Tschirschnitz M, Ring J (1993). Plasma concentrations of arginine vasopressin, oxytocin and angiotensin in patients with hymenoptera venom anaphylaxis. Regul Pept.

[10] Shimamoto K, Nakagawa M, Iimura O (1990). *In vivo* concentrations of kinins and angiotensins. Horm Metab Res Suppl.

[11] Sim MK, Qui XS (2003). Angiotensins in plasma of hypertensive rats and human. Regul Pept.

[12] Huang W, Yang Y, Wu S, Jin Z, Bao D, Gan H (2001). Early changes of arginine vasopressin and angiotensin II in patients with acute cerebral injury. Chin J Traumatol.

[13] Brun V, Masselon C, Garin J, Dupuis A (2009). Isotope dilution strategies for absolute quantitative proteomics. J Proteomics.

[14] Sturm R, Sheynkman G, Booth C, Smith LM, Pedersen JA, Li L (2012). Absolute quantification of prion protein (90–231) using stable isotope-labeled chymotryptic peptide standards in a LC-MRM AQUA workflow. J Am Soc Mass Spectrom.

[15] Chambers EE, Fountain KJ, Smith N, Ashraf L, Karalliedde J, Cowan D, Legido-Quigley C (2014). Multidimensional LC-MS/MS enables simultaneous quantification of intact human insulin and five recombinant analogs in human plasma. Anal Chem.

[16] Thomas A, Geyer H, Kamber M, Schänzer W, Thevis M (2008). Mass spectrometric determination of gonadotrophin-releasing hormone (GnRH) in human urine for doping control purposes by means of LC-ESI-MS/MS. J Mass Spectrom.

